# Mitochondrial dysfunction in diabetic nephropathy: insights and therapeutic avenues from traditional Chinese medicine

**DOI:** 10.3389/fendo.2024.1429420

**Published:** 2024-07-23

**Authors:** Dan-mai Zhao, Rui Zhong, Xiao-tian Wang, Zhong-hong Yan

**Affiliations:** Heilongjiang University of Traditional Chinese Medicine, Harbin, Heilongjiang, China

**Keywords:** diabetic nephropathy, mitochondrial dysfunction, traditional Chinese medicine, oxidative stress, mitophagy

## Abstract

Diabetic nephropathy (DN) is a microvascular complication of diabetes mellitus. The progressive damage to glomeruli, tubules, and interstitium in the kidneys can lead to the development of chronic kidney disease (CKD) and end-stage renal disease (ESRD). Most of the energy we need comes from mitochondria. Mitochondria are best known as the sites for production of respiratory ATP and are essential for eukaryotic life. The pathogenesis of DN involves a variety of factors, such as altered haemodynamics, oxidative stress, and inflammation, and studies from animal models suggest that mitochondrial dysfunction plays an important role in the development of DN. Traditional Chinese medicine (TCM) has a history of more than 2,500 years and has rich experience and remarkable efficacy in the treatment of DN. Recent studies have found that TCM may have great potential in regulating mitochondrial dysfunction in the treatment of DN. This review will elucidate the main causes of mitochondrial dysfunction and the relationship with DN, and explore in depth the potential mechanisms of TCM to protect the kidney by improving mitochondrial dysfunction. Current pharmacological treatments for patients with DN do not prevent the inevitable progression to ESRD. With the rich variety of Chinese herbs, TCM is expected to be the most promising candidate for the treatment of DN as we continue to learn more about the mechanisms of DN and incorporate the current advances in extraction techniques.

## 1 Introduction

Diabetic nephropathy (DN) is a chronic disease stemming from diabetes mellitus, characterized by microangiopathy and structural and functional renal alterations, standing as the foremost cause of chronic kidney disease (CKD) and end-stage renal disease (ESRD) (1). Epidemiological data reveals a global prevalence of diabetes mellitus among individuals aged 20-79 years, reaching 537 million in 2021, with projections indicating a rise to 784 million individuals (2). It is estimated that 30-40% of diabetic patients will eventually develop DN (3). Initial stages of DN manifest with glomerular hyperfiltration and hypertrophy, later progressing to impaired glomerular filtration barrier, heightened urinary albumin excretion, and accumulation of excessive extracellular matrix (ECM), leading to thylakoid stroma accumulation, hypertrophy, and consequent nodular glomerulosclerosis and interstitial fibrosis, culminating in ESRD and renal failure (4, 5). Currently, the traditional treatment of DN is mainly based on weight reduction, blood pressure and glucose control, and the use of renin-angiotensin system inhibitors (RASIs), including angiotensin-converting enzyme inhibitors (ACEIs) or angiotensin receptor blockers (ARBs) as the first-line medication for DN (6). However, there are some side effects associated with long-term use of these medications, such as ACEIs may cause angioedema and ARBs have the risk of causing hyperkalemia (7, 8). This not only substantially diminishes patients’ quality of life but also imposes significant burdens on families and society. Hence, the quest for novel therapeutic strategies or drugs to address diabetic nephropathy or mitigate disease advancement is imperative.

Mitochondria, the double-layered membrane organelles renowned as the “powerhouses” of the cell, provide the main energy for cellular activities through oxidative phosphorylation(OXPHOS) (9). The kidney, as a metabolic organ in the human body, requires a large amount of ATP to maintain its normal function, and the oxygen consumption and mitochondrial content of the kidneys are second only to the heart (10). Mitochondrial dysfunction is closely related to the development of DN, and the loss of mitochondrial control affects renal health (11). Activation of mitochondrial autophagy by maintaining mitochondria-associated endoplasmic reticulum membrane(MAM) integrity can attenuate renal tubular injury in DN during the treatment of DN (12). Under diabetic conditions, MAMs in podocytes are increased, leading to excessive mitochondrial fission and resulting in podocyte injury (13). However, excessive mitochondrial fusion may also be involved in the pathogenesis of DN (14). As revealed by the detection of mitochondrial DNA(mtDNA)in blood samples from DN patients, systemic mitochondrial dysfunction initiated by glucose induced mtDNA damage may be involved in the development of DN (15). Diabetes mellitus is known as “xiaoke” in traditional Chinese medicine and is characterised by polydipsia, polyuria, and emaciation. DN falls under the category of “xiaoke kidney disease” in TCM (16). With a history spanning thousands of years, TCM is an integral component of China’s healthcare system, focusing on the harmonization of yin and yang, and the kidneys are considered the foundation of “*Yin and Yang* of the five *zang* viscera”. According to TCM principles, the kidney governing water metabolism, emphasizing that urine production and excretion rely on the kidney qi transpiration and gasification (17). In TCM, the screening and prediction of active ingredients, disease genes, and related proteins have been conducted. Through some digital mining and analysis techniques, such as network pharmacology and molecular docking technology, the active ingredients of drugs, disease genes and related proteins are screened and predicted (18, 19). This approach aligns with the TCM concept of treating DN through “multi-components, multi-targets, and multi-pathways.” Consequently, research efforts have shifted from evaluating the clinical efficacy of single empirical prescriptions to exploring the active ingredients of TCM and the mechanisms underlying the combinations of Chinese herbal medicines, offering new therapeutic prospects for a diverse patient population.

This review discusses and analyses the relationship between mitochondrial dysfunction (e.g., mtDNA damage, mitochondrial respiratory chain damaged, mitochondrial dynamics disorders, impaired mitophagy, elevated ROS and the development of oxidative stress) and DN. It summarizes the most recent discoveries in Chinese medical therapies for DN, emphasizing their impact on regulating mitochondrial dysfunction, aiming to serve as a guide for the prevention and treatment of DN. Reviewed literature from PubMed, Web of science and China National Knowledge Infrastructure using key terminologies related to DN and Mitochondrial Dysfunction.

## 2 Mitochondrial dysfunction and DN

**Figure 1:**
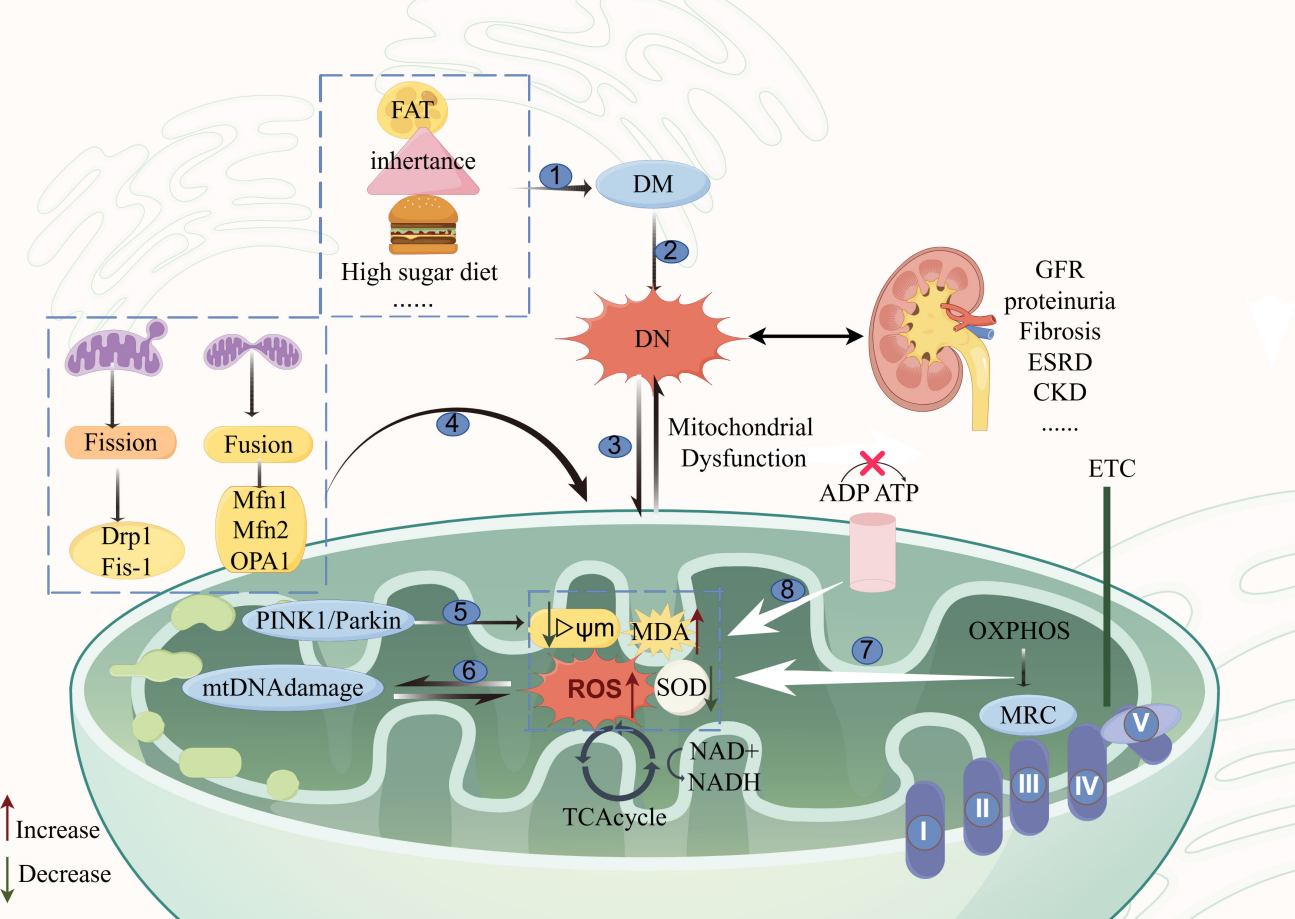
Diagram of the mechanism of action of mitochondrial dysfunction and DN. The main causes of mitochondrial dysfunction and the main pathways affecting the development of DN are as follows: ①: etiologies of diabetes mellitus; ②: the onset of diabetic nephropathy is rooted in diabetes mellitus; ③: mitochondrial dysfunction and diabetic nephropathy are mutually interactive; ④: mitochondrial kinetic aberrations; ⑤: mitophagy signaling pathway; ⑥: mitochondrial DNA damage pathway; ⑦: disorders of the mitochondrial respiratory chain; ⑧: impairment in ATP conversion. The figure was drawn using Figdraw (www.figdraw.com). Mfn1, Mfn2, mitochondrial fusion proteins 1 and 2; OPA1, optic atrophy protein 1; Fis1: receptor proteins mitochondrial fission1; Drp1, mitochondrial dynamin-related protein 1; MRC, The mitochondrial respiratory chain; OXPHOS, oxidative phosphorylation; DN, Diabetic nephropathy; DM, Diabetes mellitus; ROS, Reactive Oxygen Species; MDA, malondialdehyde; TCAcycle, The tricarboxylic acid cycle; GFR, Evaluation of glomerular filtration rate; ESRD, end-stage renal disease; CKD, chronic kidney disease.

The development of DN is closely associated with damage to mtDNA, mitochondrial respiratory chain disorders, mitochondrial dynamics disorders, mitophagy damage, and elevated mitochondrial derivative ROS with the development of oxidative stress. The main mechanisms of action of mitochondrial dysfunction and DN are shown in **Figure 1**.

### 2.1 Mitochondrial DNA damage and DN

Mitochondria contain their own genetic material, known as mitochondrial DNA (mtDNA), which encodes key proteins in the mitochondrial respiratory complex (20). This circular double-stranded DNA molecule is comprised of heavy and light strands, encoding a crucial array of proteins essential for the mitochondrial respiratory complex (21). The coordination between the mitochondrial genome (mtDNA) and the nuclear genome (nDNA) is essential for regulating mitochondrial function. Unlike nDNA, mtDNA is closer to sites of phosphorylated oxidation and lacks protection from the abundant histones in mitochondria, making it susceptible to damage from exogenous and endogenous stresses. mtDNA damage consists of five types: alkylation damage, hydrolysis damage, adduct formation, base mismatches, and DNA strand breaks (22). mtDNA, which is usually located in the mitochondrial matrix in the vicinity of major sites of reactive oxygen species (ROS) generation, not only induces oxidative damage, but also its mtDNA fragments escape from the matrix to the extracellular compartment and activate downstream inflammatory responses (23). Recent studies highlight the mitochondrial matrix as a principal site of mtDNA damage.

In renal cells, disruption of mitochondrial integrity may lead to the release of the mtDNA gene into the urine, potentially serving as a surrogate marker for renal mitochondrial dysfunction. Studies investigating the relationship between mtDNA copy number and the risk of kidney disease progression have revealed a negative correlation between serum mtDNA copy number levels and the progression of CKD and renal failure in individuals of both black and white ethnicities with mild to moderate CKD, as well as in patients with diabetes mellitus, and patients with albuminuria (24). In a case-control study, it was observed that 50 healthy participants, 50 patients with type 2 diabetes mellitus, and 50 patients with diabetic nephropathy had lower mtDNA copy numbers than those with diabetes mellitus and healthy controls, which further suggests that the mtDNA copy number in peripheral blood could serve as a novel potential biomarker for diabetic nephropathy patients (25). These findings collectively underscore the significance of mtDNA damage on diabetes and kidney disease, emphasizing the potential utility by detecting mtDNA in assessing and predicting diabetic nephropathy.

### 2.2 Mitochondrial respiratory chain damaged and DN

The mitochondrial respiratory chain (MRC) is the basic structure of OXPHOS and occupies a central position in cellular energy metabolism. Comprising two mobile electron carriers, ubiquinone and cytochrome C, as well as four multiprotein enzyme complexes (complexes I-IV) (26). These enzyme complexes facilitate the phosphorylation of adenosine diphosphate (ADP) to ATP by harnessing an electrochemical gradient across the inner membrane via an electron transport chain (27).

The kidney, being a highly metabolic organ rich in mitochondria, demands substantial ATP to sustain its physiological functions. Damage to the mitochondrial respiratory chain impedes electron transmission and reduces ATP synthesis. Consequently, impaired ATP synthesis disrupts the cation gradient, allowing Ca2+ influx, which in turn triggers the breakdown of mitochondrial membrane phospholipids. This process increases mitochondrial membrane permeability, leading to the release of the cytochrome C into the cytosol, thereby accelerating the initiation of apoptosis initiation and ultimately decreasing the mitochondrial membrane potential (MMP) (28).

Moreover, the MRC is a major site for ROS production, and in the presence of hyperglycaemia, damaged mitochondria produce excessive electron leakage in MRC complex I and at the interface between MRC complex III and coenzyme Q (29). The escaped electrons are then individually reduced, giving rise to oxygen free radicals (OFRs) that subsequently generate deleterious hydroxyl radicals, culminating in ROS accumulation; The excessive ROS production from impaired MRCs exacerbates mtDNA and respiratory chain complex enzymes, further intensifying mitochondrial damage (30). Previous studies have revealed a reduction in 31 mitochondrial proteins podocytes cultured under high glucose conditions, with key components of complexes III, IV, and V experiencing diminished levels at the respiratory chain (31). Studies have found a significant increase in the expression of respiratory chain complex subunits CO1, CO2, CO3, ATP6 and ATP8 in repair experiments of renal tubular epithelial cells by investigating lipocalin (32). Currently, some researchers also consider the MRC as a potential therapeutic target for chronic renal failure, underscoring its pivotal role in renal injury progression (33). The aforementioned studies indicate that renal mitochondria are particularly vulnerable to impairment in a high-glucose milieu, where the concurrent overproduction of ROS and diminished ATP synthesis exacerbate the progression of DN upon MRC injury.

### 2.3 Mitochondrial dynamics disorders and DN

The process of changing mitochondrial morphology, location and quantity within eukaryotic cells is tremed as mitochondrial dynamics, which maintains a dynamic equilibrium through constant fusion and fission (34). When there is an imbalance between these processes, it is referred to as a mitochondrial kinetic disorder. Mixing and exchange of contents between mitochondria is facilitated by mitochondrial fusion, allowing rapid metabolite exchange to replenish damaged mitochondria and maintain mitochondrial function (35). The three large kinetic protein family GTPases responsible for mitochondrial fusion are mitochondrial fusion proteins 1 and 2 (Mfn1, Mfn2), and optic atrophy protein 1 (OPA1). Mfn1 and Mfn2 are responsible for the fusion of the outer mitochondrial membrane, and the fusion of the inner mitochondrial membrane is mediated by OPA1; mitochondrial fission splits to form two new organelles, thereby providing a sufficient number of mitochondria for cell growth by providing a sufficient number of mitochondria; damaged or dysfunctional mitochondria can also be eliminated by fission to control mitochondrial mass and promote apoptosis. Mitochondrial fission is regulated by the receptor proteins mitochondrial fission1(Fis1), mitochondrial fission factor (Mff), and mitochondrial dynamin-related protein 1 (Drp1), and fission occurs at the contact site between the mitochondria and the endoplasmic reticulum at the contact site (36–38). In diabetes mellitus, insulin secretion disorders and elevated blood glucose cause an imbalance between cellular energy and demand. To maintain normal cellular energy balance, mitochondria down-regulate fusion proteins and up-regulate fission proteins, leading to an increase in the number of mitochondria, often resulting in small, fragmented, and non-functional mitochondria. As the disease progresses, this compensatory mechanism becomes insufficient, leading to secondary dysfunctions such as diabetic nephropathy (39).

It was discovered that highly fragmented and discrete mitochondria were present in high glucose-induced human podocytes, which cover the outer side of glomerular capillaries and are essential for the glomerular filtration barrier. Decreased expression of Opa1, Mfn1, and Mfn2, along with increased expression of Drp1 in podocytes cultured under high glucose conditions, mitigated excessive mitochondrial fission and cellular damage in podocytes when thromboxane/prostaglandin receptor (TP receptor) was silenced (40). Calcium/calmodulin-dependent protein kinase type 1 (CAMK1) was found to alleviate the progression of DN by regulating mitochondrial dynamics in a mouse model of DN. Conditional deletion of CAMK1 promoted mitochondrial fission and exacerbated renal injury in diabetic mice, while *in vitro* studies found that overexpression of CAMK1 inhibited mitochondrial fission and mitigated high-glucose-induced apoptosis in HK-2 cells (41). In another experiment (13), it was revealed that the mitochondria-associated endoplasmic reticulum membrane (MAM) promotes mitochondrial fission in podocytes under high-glucose conditions via the AKAP1-Drp1 pathway, and that excessive mitochondrial fission in podocytes is a key feature of DN. A-kinase anchoring protein 1 (AKAP1) is located in MAM, and knockdown of AKAP1 significantly reduces mitochondrial fission and attenuates high glucose-induced podocyte injury by regulating the phosphorylation of Drp1 and subsequent mitochondrial translocation. Ubiquitinated degradation of Mnf2 was found to promote ischemia-reperfusion-induced apoptosis of renal tubular epithelial cells in an animal model, suggesting that reduced mitochondrial fusion is closely associated with acute kidney injury (42). There is increasing evidence that mitochondrial fusion and fission can impact podocytes, renal tubular epithelial cells, and others in a high-glucose environment, and that an abnormal kinetic balance of mitochondria severely influences the progression of DN.

### 2.4 Mitophagy and DN

The process of mitophagy involves the selective encapsulation and degradation of damaged or dysfunctional mitochondria by cells through the autophagy mechanism, thereby maintaining mitochondrial and intracellular homeostasis (43). Mitophagy begins with the depolarization and lose of membrane potential in damaged mitochondria, which are then engulfed by autophagosomes to form mitochondrial autophagosomes. These structures are subsequently delivered to lysosomes for degradation, ultimately leading to the recycling of mitochondrial contents (44). Ubiquitin-mediated mitophagy, which includes PTEN-induced putative kinase 1 (PINK1)-dependent and Parkin dependent mitophagy pathways, is the major mitophagy pathway in mammals (45). PINK1 undergoes transfer to the inner mitochondrial membrane (TIM23) complex with the help of the outer mitochondrial membrane (TOM22, TOM70, TOM20). Once there, PINK1 is cleaved by the mitochondrial processing peptidase (MPP) and the presenilin-associated rhomboid-like (PARL), and then degraded via the N-end rule pathway. When mitochondria are damaged, the C-terminus in PINK1 binds to TOM7, leading to the stable accumulation of PINK1 in the mitochondrial outer membrane and trans-autophosphorylation. Parkin is an E3 ubiquitin ligase, Parkin acts as a signal amplifier which is readily phosphorylated and activated by PINK1 at Ser65. Activated Parkin ubiquitinates many mitochondrial outer membrane proteins, and these ubiquitinated proteins are further phosphorylated by PINK1, which recruits more Parkin into the mitochondria, resulting in the production of more ubiquitin chains (46, 47). Polyubiquitin chains lead to phagocytosis of target mitochondria within autophagosomes by binding to the receptor and to the microtubule-associated protein LC3 on the autophagosome membrane (48). In addition, ubiquitin-independent pathways of autophagy can initiate mitophagy without ubiquitination by binding directly to L3. For example, Nip3-like protein X (NIX)/BCL2-interacting protein 3-like (BNIP3L) receptor, BCL2-interacting protein 3 (BNIP3) receptor, FUN14 domain containing 1 (FUNDC1) receptor, and others (49). Thus, if mitophagy is impaired, there is an increased accumulation of dysfunctional mitochondria, which may lead to abnormal cellular function and contribute to the development of DN.

In a study of DN, the aldose reduction inhibitor WJ-39 demonstrated efficacy in improving renal tubular morphology and inhibiting renal fibrosis in DN rats by inhibiting the activation of the polyol pathway. Its mechanism of action appears to be linked to the activation of the PINK1/Parkin pathway, promoting mitophagy and attenuating apoptotic processes. This was corroborated by experiments on HK-2 cells exposed to high glucose conditions, confirming that WJ-39’s tubular protection is closely tied to enhanced mitophagy via PINK1/Parkin signalling pathway (50). Similarly, by detecting the effect of hypoxia-inducible factor-1 alpha (HIF-1α) on mitophagy -associated proteins in HK-2 cells cultured under high glucose conditions, revealed that HIF-1α activated the parkin/PINK1 signalling pathway. HIF-1α-Parkin/PINK1-mediated mitophagy prevented apoptosis and ROS production in HG-exposed HK-2 cells, which could protect renal tubular cells from apoptosis and ROS (51). Furthermore, in a canine model of type 1 DN treated with N-acetylcysteine (NAC) and insulin, a reduction in the gene expression levels of renal autophagy receptors BNIP3, NIX, and FUNDC1 was observed, alongside facilitation of mitochondrial fusion and inhibition of fission (52). Maintaining autophagic homeostasis is vital for normal renal function, and it has been noted that mitophagy occurs in DN in various renal cells, including podocytes, glomerular mesangial cells, and proximal tubular epithelial cells. Herbs and active compounds have shown promise in reducing renal injury by modulating mitophagy in DN (53). The above suggests that the buildup of apoptotic material is susceptible to oxidative stress and inflammatory reactions, emphasizing the crucial role of promptly eliminating damaged mitochondria and apoptotic cells to cellular homeostasis.

### 2.5 Increase in mitochondria-derived ROS and oxidative stress in DN

Oxidative stress (OS) is a metabolic dysfunction mediated by an imbalance between the overexpression of reactive oxygen species (ROS) and the body’s antioxidant defence system (54). The mitochondrial respiratory chain primarily generates ROS, with a minor amount produced by OXPHOS during ATP generation. ROS, as a by-product of OXPHOS, can play an important role in maintaining redox homeostasis, signal transduction, and defence against infections. The most common ROS include superoxide anion (O_2_^-^), hydrogen peroxide (H_2_O_2_), and hydroxyl radicals (OH •). OS results when ROS production exceeds the scavenging capacity of the antioxidant defence system (55). Abnormalities in oxidative stress metabolic pathways are involved in the production of diabetic free radicals and complications, including glucose oxidation and activation of glyceraldehyde-3-phosphate dehydrogenase, increased expression of advanced glycosylation end-products (AGEs) and their activating ligand receptors, diacylglycerol formation and activation of protein kinase C isoforms, overactivation of the hexosamine pathway, and increased flux of the polyol pathway (56). These aberrant pathways induce mitochondrial dysfunction and OS. In a high-glucose environment, mitochondrial OS impairs energy metabolism, activates inflammatory pathways, enhances inflammatory responses, and induces apoptosis by disrupting normal cellular metabolism (57). Experiments in DN rat model have shown significant mitochondrial OS damage in rats with DN, the mechanism of which is associated with the downregulation of PGC-1α-SIRT3-SOD_2_ and increased ROS activity (58). It was found that Salvianolic acid salt (SAL) reduced 8-hydroxy-2 deoxyguanosine (8-OHdG) with MDA levels by inhibiting NOX4-based NADPH oxidase in db/db mice. Its main active ingredient, SalB, blocked high glucose-induced ROS production and apoptosis in NOX4-based human podocytes thereby ameliorating oxidative stress and podocyte damage (59). All of the above studies illustrate that mitochondrial mitochondria-derived ROS constitute a primary source of oxidative stress in the kidney, emphasizing the significance of reducing ROS generation as a pivotal therapeutic strategy for DN.

## 3 TCM targeted mitochondrial therapy DN

TCM employs a nuanced approach to healing, harnessing the intricate pharmacological properties of natural flora. Through meticulous concoction and processing, these plants are transformed into formulas or their active constituents are extracted and isolated. In our discussion, we explore how Chinese herbal formulas, Chinese patent medicine, and compounds and extracts from Chinese herbs are utilized to regulate mitochondrial function in the treatment of DN.

### 3.1 Chinese herbal formulas

Chinese herbal formulas consist of two or more herbs that act synergistically and reduce toxicity. Herbal formulas have a long history of treating diabetic nephropathy, and this chapter summarises the potential link between the improvement of DN by certain herbal formulas and the modulation of mitochondrial dysfunction (**Table 1**).

**Table 1:** Mechanism of action of herbal formulations.

Chinese herbal formulas	Composition	Experiment Model	Mechanism of action	Effect	Ref.
Huangqi-Danshen decoction	*Astragali Radix ,Salviae Miltiorrhizae Radix et Rhizoma*	STZ-induced db/db mice	Drp-1↓,PINK1↓,Parkin↓	Reduced blood glucose, improved renal function, alleviated renal injury; Reduced mitochondrial fission; Inhibited excessive mitophagy;	(60)
JinChanYiShen TongLuoFormula	*Cordyceps cicadae,Radix astragali,Radix scrophulariae,Rhizoma curcumae,Bombyx batryticatus,Hirudo,twigs of winged Euonymus,Radix cyathulae*	STZ-induced DN in SD rats	the activity of mitochondrial respiratory chain complexes I, III, and IV↑,Bax↓,C-Caspase3↓,apoptosis ratio↓	Reduced proteinuria and improved renal function; Reduced apoptosis; Improved mitochondrial dysfunction	(61)
		HG induced HK-2 cells	MMP↑,the activity of mitochondrial respiratory chain complexes I, III, and IV↑,Bax↓,Bcl-2↑,PINK1↑,Parkin↑,LC3-II↑,P62↓,HIF-1α↑	Reduced apoptosis; Enhanced mitophagy; Ameliorated mitochondrial dysfunction	
Huangqi decoction	*Astragalus,Poria,Trichosanthes,Ophiopogon ,Schisandra,Licorice,Rehmannia*	STZ-induced Male C57BL/6J mice	Nephrin↑, WT1↑,Nox4↓,gp91phox↓,rac-1↓,Nox4/p53/Bax↓	Improved kidney function and reduced kidney damage; Reduced oxidative stress;	(62)
		HG induced diabetic SD rats podocytes	Nephri,NADPH oxidase activity↓ ,ROS↓,Nox4↓,gp91phox↓,rac-1 mRNA↓, Bax↓,Cytc↓,Bcl-2↓, Bcl-xl↓, cleaved RAPR-1↓, cleaved caspase 9↓ ,caspase 3↓	Reduced podocyte apoptosis; Inhibited oxidative stress; Improved mitochondrial dysfunction	
Danggui Buxue decoction	*Astragali Radix,Angelicae sinensis radox*	STZ-induced DN in SD rats	ROS↓,Synaptopodin↑,Podocin↑,cleaved Caspase-3↓,AKAP1,p-Drp1(Ser637)↓,Mfn2,Bcl-2/BAX↑	Reduced hyperglycemia and improved kidney function; Alleviated mitochondrial damage; Inhibited podocyte apoptosis	(63)
XiaoYuXieZhuo decoction	*Astragali Radix,Cyathulae Radix,Pheretima,Persicae Semen,Rhei radix et rhizoma,Plantago asiatica L*	db/db mice	HIF1α mRNA↓,nephrin mRNA↑	Improved renal function, reduced renal pathological damage; Reduced podocyte apoptosis	(64)
TongLuoYiShen formula	*Rehmannia glutinosa,Cornus officinalis,Dioscoreae Rhizoma,Poria,Aconitum carmichaeli Debx,Rhei Radix et Rhizoma,Persicae Semen,Scorpio,Piscicola,Alismatis Rhizoma*	STZ-induced DN in SD rats	TFAM,PGC-1α mRNA↑,MMP↑	Improved renal function; Reduced mitochondrial damage in podocytes;	(65)

“↑” represents the up-regulated targets, “↓” represents the down-regulated targets.

Huangqi Danshen decoction (HDD) comprises *Astragali Radix* (Huang-qi) and *Salviae Miltiorrhizae Radix et Rhizoma* (Dan-shen). The extracts of both components can suppress Drp-1 expression and regulate the PINK1/Parkin pathway. This modulation leads to an improvement in mitophagy, which in turn alleviate renal injury in DN mice. Phenotyping experiments revealed that glomerular hypertrophy as well as the increase of thylakoid cells and thylakoid matrix were significantly improved in mice, and extensive fusion of pedunculated synapses was ameliorated by HDD treatment in mice (60).

JinChanYiShen TongLuoFormula (JCYSTL), composed of eight Chinese herbs, can activate PINK1/Parkin-mediated mitophagy by stabilising HIF-1α. Hypoxia-inducible factor-1alpha (HIF-1α) is a key molecule that plays an important role in the hypoxic environment and can be regulated by the O2 tension of the alkaline helix-loop-helix PAS heterodimer.HIF1α- is able to attenuate renal tubular cell injury by promoting mitophagy in the HG milieu (51, 66). JCYSTL can reduce the concentrations of serum creatinine (Scr),blood urea nitrogen (BUN), uric acid (UA), and 24-h urinary albumin(UAlb)in DN rats, and increase the mitochondrial respiration. concentrations and increased the activity of mitochondrial respiratory chain complexes I, III, and IV, thereby protecting renal tubules from mitochondrial dysfunction and apoptosis under DN conditions (61).

HuangQi decoction (HQD) is a traditional Chinese herbal formula consisting of *Astragalus, Poria, Psoralea, Psidium guajava, Ophiopogonis, Schisandra chinensis, Glycyrrhiza glabra, and Radix Rehmannia*e. Clinical studies have shown that HQD can significantly improve the TCM symptoms of qi and yin deficiency in stage III DN patients, reducing the urinary albumin-creatinine ratio (UACR), blood glucose, and blood lipid levels, while improving glycolipid metabolism disorders. Moreover, HQD effectively enhances serum inflammatory factor levels, indicating promising clinical efficacy (67, 68). *In vivo* experiments revealed that HQD significantly inhibited Nox4/p53/Bax signalling and effectively alleviated progressive proteinuria, glomerulosclerosis and podocyte loss in DN mice (62). NADPH oxidase is a major source of ROS, and Nox4 overexpression enhances NADPH oxidase activity, which significantly triggers oxidative stress and activates downstream signalling pathways in the podocyte, including p53, to induce podocyte injury (69). Whether HQD can have an effect on mitochondria needs to be further verified, but it also provides a new therapeutic idea for diabetic nephropathy caused by mitochondrial dysfunction.

Danggui Buxue decoction is composed of *Astragali Radix* and *Angelicae sinensis radox* in a 5:1 dosage ratio, which has the effect of tonifying Qi and generating blood, and resolving blood stasis.It has been reported that peroxisome proliferator-activated receptor-γ coactivator-1-α (PGC-1α) is a major regulator of mitochondrial biogenesis, and it participates in mitochondrial biogenesis in renal diseases by orchestrating transcriptional mechanisms. Activation of PGC-1α also prevents renal dysfunction in various renal diseases and has emerged as a potential therapeutic target against renal aging (70, 71). Studies have shown that Danggui Buxue decoction can elevate the levels of PGC-1α, MnSOD mRNA and protein expression, reduce the levels of NLRP3, IL-1β mRNA and protein expression improve the mitochondrial dysfunction of podocytes in DN rats, alleviate oxidative stress, mitigate the inflammatory response, and slow down the progression of DN (63). Clinical trials have shown that Danggui Buxue decoction can effectively reduce blood glucose, blood lipids and urinary protein levels in patients with DN, and has the effect of protecting kidney function (72).

XiaoYuXieZhuo decoction can reduce urinary protein, improve renal function, attenuate renal structural pathological changes, and inhibit podocyte apoptosis in mice with DN by modulating HIF-1α signalling pathway (64). In a controlled clinical trial, it was found that DN patients could reduce serum leptin levels and improve clinical symptoms and renal function after taking the XiaoYuXieZhuo decoction (73). HIF-1α was found to ameliorate mitochondrial dysfunction and limit mitochondria-dependent apoptosis in DN tubular cells via Heme Oxygenase 1(HO-1). It also further ameliorated renal tubular injury in DN through HO-1 mediated control of mitochondrial dynamics. Moreover, HIF-1α attenuates high-glucose-induced renal tubular cell injury by promoting PINK1/Parkin-mediated mitophagy (51, 74).

TongLuoYiShen formula consists of ten Chinese herbs, which can enhance the mRNA expression of Peroxisome proliferator-activated receptor-γ coactivator-1-α (PGC-1α), mitochondrial transcription factor A, restore the mitochondrial membrane potential, and promote mitochondrial biosynthesis in DN rats (65). PGC-1α has been validated to play a moderate role in promoting mitochondrial generation, restoring mitochondrial membrane potential, and influencing mitochondrial quality control (75). TongLuoYiShen formula combined with candesartan can delay the renal function damage of DN patients and reduce the Chinese medicine symptoms in clinical practice, and the efficacy of the combination of Chinese and Western medicine in treating DN is remarkable (76).

All aforementioned experimental findings indicate the efficacy of Chinese herbal formulas in lowering blood glucose, reducing proteinuria, and enhancing renal function. Such formulas hold promise in the treatment of DN through the modulation of mitophagy, mitochondrial respiratory chain dysfunction, oxidative stress, and other pathways.

### 3.2 Chinese patent medicine

Chinese patent medicine(CPM) are a component of traditional Chinese medicine (TCM), and they have the advantages of ease of use, good efficacy, low dosage, and fewer side effects compared to Western pharmaceuticals (77). Certain CPM used in the treatment of DN also have an effect on mitochondrial function (**Table 2**).

**Table 2:** Mechanisms of action of CPM.

Chinese patent medicine	Composition	Experiment Model	Mechanism of action	Effect	Ref.
Gushen Jiedu capsule	Semen *Euryales,,Fructus Rosa laevigata,,Rhizome Coptis chinensis,,Rhizome Rheum tanguticum,,Radix Astragalus membranaceus , Radix Angelica sinensis*	STZ-induced DN in SD rats	podocin↑ , nephrin ↑,Bax ↓,BCL-2↑,p-Akt ↓,	Reduced proteinuria; ImproveD renal function; Reduced apoptosis; Regulated mitochondrial apoptosis	(78)
QiDiTangShen granules	*Astragali Radix,Rehmanniae radix praeparata,Euryale Semen,Sophorae Tonkinensis Radix et Rhizoma,Hirudo ,Rhei Radix et Rhizoma,Baihuasheshecao*	STZ-induced DN db/db mice	TGF-β↓,α-SMA↓ , Col I↓,autophagic vacuoles ↑ATG14 ↑,beclin1↑,LC3-II↑,p62↓,p-AMPK (ser172)/total AMPK↑,SIRT1↑,p-mTORC1 (ser2448)/total mTOR↓	Reduced proteinuria; Ameliorated renal fibrosis; Modulation of nutrient-sensing signalling pathways that activate mitophagy. Alleviated mitochondrial damage	(79)
Yishen capsule	*Astragalus membranaceus,,Angelica sinensis,,Euryale ferox,Alisma orientalis, Rhodiola rosea*	STZ-induced DN in SD rats	SIRT1↑,Beclin-1↑ ,LC3-II ↑,NF-κBp65 ↓,	Reducted proteinuria; Ameliorated renal pathological damage;	(80)
		STZ-induced diabetic SD rats podocytes	nephrin ↑,NF-κB p65 ↓,nuclear translocation of SIRT1 ↑,Beclin-1↑ ,LC3-II ↑	Promoted mitophagy; Ameliorated podocyte damage	
San-Huang-Yi-Shen capsule	*Astragalus mongholicus Bunge,Cuscuta chinensis Lam,Salvia miltiorrhiza Bunge,Polygonatum kingianum Collett & Hemsl,Rehmannia glutinosa,Panax quinquefolius L.,Dioscorea oppositifolia L.,Cornus officinalis Siebold & Zucc.,Lycium barbarum L.,Euryale ferox Salisb.,Rosa laevigata Michx.,Leonurus japonicus Houtt.,Conioselinum anthriscoides ‘Chuanxiong’,Atractylodes lancea (Thunb.) DC.,Paeonia lactiflora Pall.,Gypsophila vaccaria (L.)*	STZ-induced DN in SD rats	IL-1*β* ↓,IL-6 ↓,TNF-α ↓,SOD ↑ , GSH-Px ↑,MDA↓,VDAC1↓,TOM20↓,COXIV↓,PINK1↑,Parkin↑,LC3-II↑,p62↓	Reduced proteinuria; Improved renal function; Reduced inflammation;Increased antioxidant capacity and mitophagy levels; Reduced mitochondrial damage	(81)
Danzhi Jiangtang Capsule	*Radix Pseudostellariae,Radix Rehmanniae, Cortex Moutan, Rhizoma Alismatis, Semen Cuscutae Chinensis, Leech.*	STZ-induced DN in SD rats	LPO↓,MDA↓,SOD↑,COX 2↓,iNOS↓,IL-6↓,MCP-1↓,TNF-α↓,JAK 2↓,STAT 1↓,STAT 3↓,	Improved renal function; Increased antioxidant capacity; Reduced inflammatory response;	(82)

“↑” represents the up-regulated targets, “↓” represents the down-regulated targets.

Gushen Jiedu capsule(GSJD)originated from the classic Chinese kidney tonic formula “Shuilu Erxiandan” in the Song Dynasty (1170 AD), which down-regulates pro-apoptotic protein Bcl-2-associated X protein (Bax) expression and up-regulates B cell lymphoma 2 (BCL-2) expression in the kidney of DN rats to regulate the mitochondrial apoptosis pathway. Bax and Bcl-2 are two key markers of apoptosis and play a critical role in high glucose-induced apoptosis in podocytes (83, 84). At the same time, the DN rat podocyte peduncle markers podocin and nephrin were also upregulated by GSJD, suggesting that GSJD may play a role in protecting the kidneys by modulating mitochondrial dysfunction (78).

QiDiTangShen granules (QDTS) significantly reduces urinary albumin excretion in db/db mice, improves thylakoid matrix deposition, attenuates protein and mRNA expression of Transforming growth factor β(TGF-β), α-smooth muscle actin (α-SMA) and collagen type I (ColI). It improves mitochondrial mass and the number of autophagic vesicles in renal tubular cells, while its active ingredients can have an effect on nutrient-sensitive signalling pathways, and can play a role in protecting the kidneys by activating mitophagy nutrient-sensing mitochondrial pathways (79).

Yishen capsule consists of five herbs, which modulate silent information regulator sirtuin 1 (SIRT1) and acetylated nuclear factor kappa-light-chain-enhancer of activated B cells(NF-kB) p65, increase the expression of SIRT1, LC3-II, and Beclin-1 *in vitro* and *in vivo*, thereby enhancing the autophagy process in podocytes through the SIRT1/NF-κB pathway, ultimately ameliorating DN (80). In addition, Yishen capsules have demonstrated efficacy in alleviating the symptoms of diabetic nephropathy by modulating the NOD-like receptor signalling pathway (85).

San-Huang-Yi-Shen capsule (SHYS) has demonstrated clinical efficacy in reducing urinary albumin levels in patients with DN, markedly enhancing renal function and mitigating renal pathological damage, with early intervention proving to be significantly more effective (86). PINK1/Parkin-mediated mitophagy is closely related to diabetic nephropathy. It has been shown that activation of PINK1/Parkin-mediated mitophagy significantly improves mitochondrial function in renal tissues, thereby attenuating renal injury in DN (87). Superoxide dismutase (SOD), catalase (CAT), and glutathione peroxidase (GPX) are antioxidant enzymes necessary for scavenging ROS from various cellular compartments and responding to stressful situations (88). SHYS capsule effectively improved renal function and reduced insulin resistance, and also up-regulated the levels of SOD, GSH-Px, reduced MDA production, and improved antioxidant capacity. It also promoted PINK1/Parkin-mediated mitophagy and inhibited the activation of NLRP3 inflammatory vesicles, thus improving mitochondrial damage and inflammatory response (81).

Danzhi Jiangtang Capsule(DJC)is a traditional Chinese medicine formula, which consists of five Chinese medicines: *Radix Pseudostellariae,Radix Rehmanniae, Cortex Moutan, Rhizoma Alismatis,Semen Cuscutae Chinensis and Leech*. Malondialdehyde (MDA) is one of the end products of membrane lipid peroxidation (LPO), which is considered a biomarker of oxidative stress, induced by excess ROS. The kidneys are rich in mitochondria, which are more susceptible to damage from oxidative stress (89, 90). DJC reduces LPO and MDA, increases SOD, and improves the antioxidant capacity of the kidneys. DJC treatment significantly reversed JAK2-STAT1/STAT 3 activation and ameliorated STZ-induced renal inflammatory response by inhibiting the JAK-STAT signalling pathway (82).

The results of the above studies suggest that CPM contributes to diminishing podocyte apoptosis, ameliorating thylakoid matrix deposition, and mitigating renal tubular injury. The mechanism is associated with the enhancement of mitophagy and the attenuation of oxidative stress, thereby reducing the inflammatory response.

### 3.3 Compounds and extracts in herbal medicine

This section encapsulates a variety of Chinese herbal compounds and extracts that exhibit promising therapeutic effects on DN, potentially through their action on mitochondria (**Table 3**).

**Table 3:** Mechanisms of action of compounds and extracts in herbal medicine.

Category	Extractive compound of Chinese herbal medicine	Experiment Model	Mechanism of action	Effect	Ref.
terpenes	Andrographolide	STZ-induced male C57BL/6 mice	α-SMA↓,FN↓,collagen |↓ collagen type IV↓ KIM-1↓,E-cadherin↑,cleaved-caspase-3↓,Bcl2 mRNA↑,Bax mRNA↓,Mfn2↑,Fis1↓,NLRP3↓,caspase-1↓,IL-1β↓	Reduced renal tubular interstitial damage and fibrosis; Improved mitochondrial function; Inhibited renal tubular interstitial inflammation;	(91)
		HK-2 cells under HG treatment	α-SMA↓,FN↓,KIM-1↓,caspase-3↓,Bax↓,cleaved-caspase-3↓,Mfn2↑,Fis1↓,mtROS↑,NLRP3↓,caspase-1↓,IL-1β↓	Protected renal tubular epithelial cells; Improved mitochondrial function Reduced ROS; Reduced renal tubular inflammation	
	Ginsenoside	STZ-induced DN in SD rats	NF-α↓,IL-6↓,MDA↓,SOD↑, p-PI3K↑,p-AKT ↑	Attenuated renal injury; Inhibited inflammatory; Reduced oxidative stress;	(92)
		HG-treated HBZY-1 cells	Bcl-2↑,Bax↓, cleaved caspase-3↓,cleaved caspase-9↓,TNF-α↓,IL-1β↓, IL-6 ↓,MDA ↓, ROS ↓,LDH ↓,SOD↑,GSH-Px↑,PI3K/AKT↑,FOXO3 ↓	Reduced apoptosis; Mitigated oxidative stress Reduced inflammatory response	
	Astragaloside II	STZ-induced DN in SD rats	nephrin↓,c-caspase-3↓,podocyte apoptosis↓,Mfn 2↑, Fis 1↓, P62↓,LC3-II/LC3-I↑;Nrf2↑,PINK1-Parkin↑,Keap 1↓	Attenuated podocyte apoptosis in diabetic rats; Restored mitochondrial morphological changes and autophagy; Ameliorated mitochondrial dysfunction	(93)
steroidal saponins	Diosgenin	STZ-induced DN in SD rats	NOX4↓, complex I–V of MRC↑,Bax↓,CytC↓,Apaf-1↓,cleaved caspase 3↓,cleaved caspase 9↓,Bcl 2↑,	Alleviated renal injury in DN rats; Inhibition of mitochondrial apoptosis and apoptosis in DN rats	(94)
		HK-2 cells under HG treatment	ROS↓,NDUFA4↑, SDHA↑,UQCRC2↑,COX17↑,ATP6↑,NOX4↓,MtMP,Apaf-1↓,cleaved caspase 3↓,cleaved caspase 9↓,Bcl 2↑	Reduction of ROS levels; Improvement of mitochondrial respiratory chain disorders; Reduction of apoptosis, improvement of mitochondrial dysfunction	
flavonoids	Formononetin	STZ-induced DN in SD rats	Bcl-2 ↑,Bax↓,c-caspase-3↓,Drp1↓ , Fis1 ↓, Mfn2↑,Sirt1↑,PGC-1α↑	Improvement of renal function and alleviation of renal tubular injury; Reduced apoptosis; Reduced renal mitochondrial morphological and kinetic abnormalities	(95)
		HK-2 cells under HG treatment	Bcl-2 ↑,Bax↓,c-caspase-3↓,MMP↑,ROS↓,mitochondrial superoxide ↓,Drp1↓, Fis1 ↓, Mfn2↑,Sirt1↑,PGC-1α↑	Reduced apoptosis; Restored mitochondrial function	
	Orientin	HG-induced MPC-5 cells	GFP-LC3 plasmid↑,LC3 ↓,p62↑,MMP↑,TOM20 colocalization↑	Protected podocyte mitochondria; Reduced apoptosis and mitophagy	(96)
	Bavachin	STZ-induced DN in db/db mice	α-SMA↓,FN↓,Col1α1↓,4-HNE↓,ROS↓,CAT ↑,HO-1 ↑,SOD2 ↑,SIRT1,NRF1↑,mtTFA ↑	Improved renal function; Reduced renal fibrosis; Reduced oxidative stress; Increased mitochondrial biosynthesis and improved mitochondrial function	(97)
		SV40-MES1 cells under high sugar treatment	ROS↓,SIRT1↑ , PGC1-α↑,α-SMA↓	Inhibited ROS production; Restoration of mitochondrial function	
lignin	SchisandrinA	STZ-induced DN in C57BL/6 mice	SOD↑,CAT↑,GHS↑,MDA↓,IL-6↓,*INF-γ*↓*,*TNF-α↓,MPT↑,GSDMD↓,GPX4↓,TXNIP↓, NLRP3↓,caspase-1↓,IL-1*β*↓,	Reduced blood glucose; Reduced proteinuria and kidney damage; Reduced oxidative stress and inflammation;	(98)
		Human glomerular endothelial cells	ROS-induced mitochondrial damage ↓, GSDMD↓,TXNIP/NLRP3↓,caspase-1↓,AdipoR1/AMPK↑,	Reduced mitochondrial damage with ROS-mediated pyroptosis; Reduced inflammatory factor release;	
	Eucommia lignans	STZ-induced DN in SD rats	IL-1β↓,TNF-α↓,MDA↓,SOD ↑,	Lower blood sugar Improved kidney function and reduced inflammation.	(99)
		HG-induced glomerular mesangial cellsglomerular	Cyto c↓, cas- pase 9 ↓, NOX4↓,ROS↓,AR↓,Nrf 2↑,HO-1↑,p-AMPK↑	Inhibited oxidative stress Reduced mitochondrial damage; Alleviated mitochondrial dysfunction.	
polyphenols	Resveratrol	STZ-induced DN in db/db mice	Drp 1↓, mitochondrial complex II and IV↑,SOD↑,GSH↑,MDA ↓, ROS ↓	Reduced proteinuria and improved renal function; Restored the morphology and function of renal cortical mitochondria in diabetic mice. Inhibited oxidative stress;	(23)
		Glomerular mesangial cells under HG treatment	Drp1↓,Ser 637phosphorylation ,PDE 4D ↓,PKA↑, Cleaved Caspase 3 ↓,cAMP↑, ROS ↓,superoxide ↓,	Inhibited mitochondrial fission; Attenuated oxidative damage and mitochondrial dysfunction; Regulated expression of phosphorylated Drp 1-Ser 637;	
extracts	Ligusticum chuanxiong rhizome	STZ-induced Male C57BL/6 mice	8-oxo-7↓,8-oxo-dG↓,TNF-α↓,ECM deposition↓,TGF-β1↓,	Improvement of renal function and reduction of renal pathological damage; Reduction of oxidative stress and inflammatory response; Reduction of renal fibrosis;	(100)
		Hepa 1c1c7 murine hepatoma cells;human breast carcinoma MDA-MB-231 cells;human renal glomerular endothelial cells (HRGEC);RAW 264.7macrophages	NO↓,Nrf2↑,NF-κB p65↓,iNOS↓,COX-2↓	Inhibited oxidative stress and reduced inflammation	
	Cassia auriculata ethanol leaf extract	STZ-induced DN in SD rats	LC3-RIPII↓,RIP-RIP 1↓,RIP-RIP 3↓,p-p38↓	Improvement of renal function and reduction of renal pathological injury; Inhibition of autophagic necrotic apoptosis	(101)
		HG-induced RGE cells	LC3-RIPII↓,RIP-RIP 1↓,RIP-RIP 3↓,p-p38↓	Inhibition of autophagic necrotic apoptosis	

“↑” represents the up-regulated targets, “↓” represents the down-regulated targets.

#### 3.3.1 Terpenes

Andrographis paniculata (AP) (Burm. f.) Wall. ex Nees, a renowned herb medicine in China. Andrographolide is the major active component of AP, exhibits diverse pharmacological activities, including anti-inflammatory, antioxidant, and antidiabetic activities (102, 103). Studies have demonstrated that Andrographolide not only inhibits extracellular matrix (ECM) over-accumulation, epithelial-mesenchymal transition (EMT) and attenuates diabetic tubular damage and fibrosis, but also prevents mesangial damage and fibrosis by regulating mitochondrial homeostasis and inhibiting mtROS overproduction and NLRP3 inflammasome activation in high-glycemic conditions (91). Moreover, pertinent research has demonstrated that Andrographolide possesses notable reproductive and nephrotoxic characteristics, markedly inhibiting the proliferation of HK-2 cells and inducing apoptosis (103). Ginsenoside Rg1 is the principal active ingredient in ginseng, the main component of ginseng root system, and is regarded as a high-quality component of ginseng root system (104). Rg1 can play a role in restoring mitophagy (105), reducing mitochondrial dysfunction (21), and ameliorating renal lipid deposition and renal fibrosis in type 2 DN mice (106). *In vitro* and *in vivo* studies demonstrated that Rg1 down-regulated MDA and ROS, up-regulated SOD, increased the expression of Bcl-2 protein, decreased the activity of proteins such as Bax, cleaved cystathionase-3, and cleaved cystathionase-9, and effectively inhibited the oxidative stress response and reduced apoptosis in rats with DN (92). Astragaloside II (AS II) is a novel saponin purified from the membranes of Astragalus membranaceus. ASII increased mitochondrial dynamics-related proteins such as Mfn 2, Fis1, P62 and LC3.ASII also increased the expression of PINK1 and Parkin, which are associated with mitophagy, in diabetic rats. By modulating the Nrf2 and PINK1 pathways, ASII improved podocyte damage and mitochondrial dysfunction in diabetic rats (93). The above results suggest that terpenoids can reduce the excessive buildup of the extracellular matrix in the kidney, alleviate renal fibrosis, and diminish apoptosis. Nevertheless, certain compounds may exhibit adverse effects, necessitating heightened vigilance during therapeutic application.

#### 3.3.2 Steroidal saponins

Diosgenin (DIO) is a plant sterol saponin, since time immemorial, DIO and its derivatives, which are obtained from the rhizomes of *Dioscorea* (popularly known as “Shanyao” in Chinese) are utilized in traditional Chinese medications (107). Diosgenin has been shown to not only alleviate the decreased HK-2 cell viability and renal pathological damage in DN rats, but also to inhibit the expression of NOX4 and restore the expression of mitochondrial respiratory chain (MRC) complex I-V, thereby reducing the production of ROS (94). The NOX family of NADPH oxidases is an important source of reactive oxygen species, of which Nox4 is most abundantly expressed in the kidney, and is therefore not only known as the renal NADPH oxidase (Renox), but also as a new therapeutic target for vascular complications of diabetes (108, 109).

#### 3.3.3 Flavonoid

Formononetin (FMN) is one of the major isoflavonoids isolated from Astragalus, which has been found to have antioxidant activity in some studies (110). It was verified by experiments that FMN attenuates DN renal tubular injury and mitochondrial damage, attenuates renal tubular apoptosis, mitochondrial fragmentation, and restores the expression of proteins related to mitochondrial dynamics and apoptosis-related proteins by regulating the Sirt1/PGC-1α pathway (95). Orientin is a flavonoid compound found mainly in Phyllostachys nigra, Trolus chinensis bunge and Passiflora. Orientin was found to inhibit oxidative stress and mitochondrial dysfunction (111). Orientin was found to restore mitochondrial autophagy in podocytes by *in vitro* experiments and has mitochondrial protective and anti-apoptotic effects (96). Bavachin is a naturally occurring flavonoid compound extracted from the seeds of the Chinese herb psoralen (112). It has been shown to be effective in anti-inflammation, inhibition of calcification and apoptosis (113, 114). Previous studies have found that Bavachin can target the electron transport chain complex to play a role in regulating mitochondrial dysfunction (115). Oral administration of Bavachin to DN mice resulted in decreased urinary microalbumin, blood urea nitrogen and creatinine clearance, and down-regulated protein levels of renal fibrosis markers α-SMA, fibronectin (FN) and collagen1α1. Meanwhile, Bavachin was further found to reduce mitochondrial ROS production and increase protein levels of antioxidant enzymes in mouse kidney tissues. Through SIRT1/PGC-1α signalling, damaged mitochondria were restored and the number of abnormal mitochondria was reduced through increased mitochondrial biosynthesis. Bavachin inhibited oxidative stress and attenuated mitochondrial dysfunction thus effectively improving DN (97). Notably, prolonged administration of low doses of Bavachin has been observed to induce renal fibrosis in zebrafish, leading to a progressive loss of the renal tubular epithelium’s normal structural specificity (116). These results suggest that exhibit significant diversity and efficacy in treating DN, potentially ameliorating mitochondrial dysfunction through various pathways, while also highlighting the necessity of considering the side effects of certain compounds during their application.

#### 3.3.4 Lignans

Schisandrin A is one of the lignans isolated from the dried fruits of Schisandra chinensis, which has a wide range of pharmacological effects in antioxidant, inhibition of apoptosis, and modulation of immunity (117, 118). It was found that Schisandrin A suppressed the levels of urea nitrogen and urinary albumin, reduced the release of inflammatory factors, inhibited the level of MDA activity, and increased the levels of SOD, CAT, and GHS in STZ-induced DN, which effectively ameliorated oxidative stress and inflammatory responses in the DN model. Meanwhile, Schisandra chinensis A attenuated iron death and NLRP3 inflammatory vesicle-mediated pyroptosis in DN via AdipoR1/AMPK-ROS/mitochondrial damage, making it a potential drug for the treatment of DN (98). *Eucommia ulmoides Oliv*, also called Du-zhong, is an old tonic herb in the traditional Chinese medicine. Lignans are derived from Eucommia ulmoides Oliver (Eucommia lignans) (119). Eucommia lignans were able to attenuate the renal inflammatory response as well as improve the antioxidant capacity in DN rats by down-regulating IL-1β, TNF-α, and MDA, and up-regulating the activity of SOD; It is known that aldose reductase(AR)is a key mediator in DN as its up-regulation promotes the progression of DN and is considered a marker of DN (120). Eucommia lignans increased the viability of glomerular thylakoid cells and down-regulated AR reversal of HG-induced mitochondrial damage in glomerular thylakoid cells (99). The aforementioned studies indicate that lignans possess considerable potential in ameliorating mitochondrial dysfunction and attenuating DN oxidative stress and inflammatory responses.

#### 3.3.5 Polyphenols

Resveratrol is a polyphenol complex that is derived from many fruits and vegetables such as peanut buds, grapes, and peanuts (121). It has great potential in the prevention and treatment of diabetes and diabetic complications (122). Phosphodiesterase-4 (PDE 4) is involved in the regulation of intracellular cyclic AMP (cAMP) levels and protein kinase A (PKA) (123). Resveratrol can ameliorate cognitive deficits in db/db mice by modulating mitochondrial function through PDE4D/PKA/Drp1 signalling (124). In recent years, it has been found that resveratrol inhibits renal mitochondrial breaks in db/db mice, restores the expression of PDE4D, PKA, phosphorylated Drp1-Ser 637, and Drp1, and attenuates the progression of DN (23).

#### 3.3.6 Herbal extracts

The rhizome of *Ligusticum chuanxiong Hort*. (Abbreviated as *LC*) has been shown to activate blood circulation and move qi in traditional Chinese medicine. Ethanol extract of Ligusticum chuanxiong rhizome (EEL) reduces oxidative stress and inflammatory response in mice; Nrf2 is considered a novel therapeutic target for DN because of its ability to eliminate ROS and regulate redox balance (125). Validated to exert antioxidant and anti-inflammatory effects in DN by *in vitro* experiments, EEL was able to inhibit oxidative stress and inflammation through the Nrf2 and NF-κB signalling pathways, exerting a renoprotective effect (100). P38MAPK is an important member of the mitogen-activated protein kinase (MAPK), which plays a pathological role in diabetes and leads to apoptosis in podocytes (126). Cassia alata ethanolic leaf extract (CALE) reduced autophagic necrotic apoptosis in diabetic nephropathy by inhibiting the expression of proteins such as LC 3-IL,IL-11,RIP-IL 1 ,p38 MAPK through *in vitro* and *in vivo* experiments, which opens up new avenues of research to study the efficacy of CALE in diabetes-related complications (101). The above studies have shown that herbal extracts can reduce renal inflammation and oxidative stress, thereby contributing to nephroprotection.

**Figure 2:**
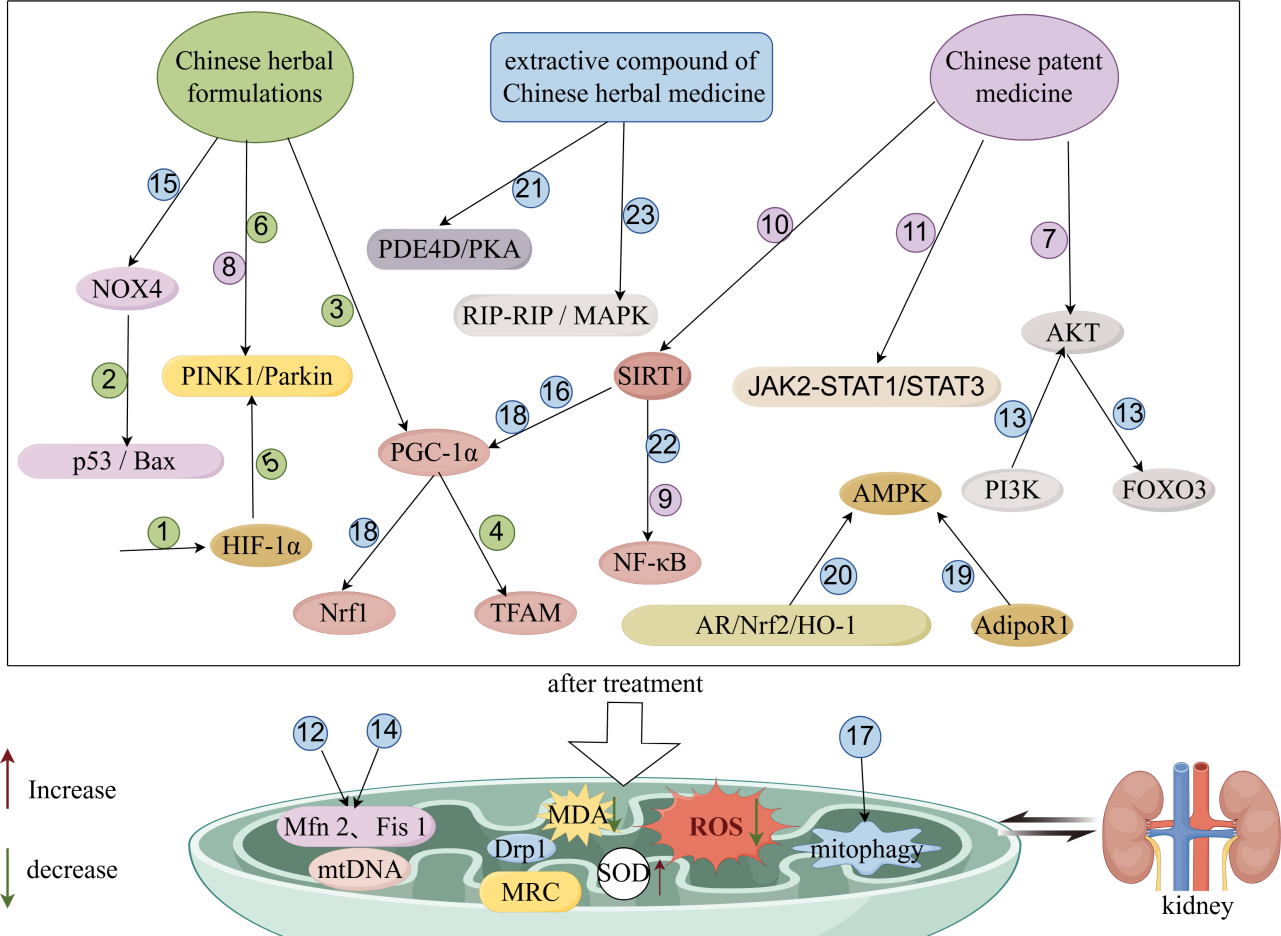
Schematic diagram of improvement of DN by TCM through modulation of mitochondrial function. The figure was drawn using Figdraw (www.figdraw.com). 1: XiaoYuXieZhuo decoction; 2: HuangQi decoction; 3: Danggui Buxue decoction; 4: TongLuoYiShen formula; 5: JinChanYiShen TongLuo Formula; 6: Huangqi Danshen decoction; 7: Gushen Jiedu capsule; 8: San-Huang-Yi-Shen capsule; 9: Yishen capsule; 10: QiDiTangShen granules; 11: DanzhiJiangtangCapsule; 12: Andrographolide; 13: GinsenosideRg1; 14: Astragaloside II; 15: Diosgenin; 16: Formononetin; 17: Orientin; 18: Bavachin; 19: SchisandrinA; 20: Eucommia lignans; 21: Resveratrol; 22: Ethanol extract of *Ligusticum chuanxiong rhizome*; 23: Cassia alata ethanolic leaf extract.

In summary, Chinese medicine (Chinese herbal formulas, Chinese patent medicine, compounds and extracts from Chinese herbs) may play a role in the treatment of DN by inhibiting the production of ROS, alleviating oxidative stress and inflammatory responses, restoring mitochondrial function, and decreasing apoptosis, thereby reducing proteinuria, improving renal function, and decreasing renal pathological damage. The main mechanism of action may be related to the pathways of PINK1/Parkin, AKAP1/Drp1, Nox4/p53/Bax, Nrf2/HO-1, SIRT1/NF-κB, JAK-STAT, Sirt1/PGC-1α, and AdipoR1/AMPK. The mechanism of action of TCM in regulating mitochondrial function to improve DN is shown in **Figure 2**.

## 4 Conclusion

With the development of society, the prevalence of diabetic nephropathy is increasing globally, which not only greatly aggravates the economic burden on society and families, but also becomes a very serious public health problem. Therefore, it is important to find new and more effective treatments with fewer side effects. Chinese medicine is a medical theory system with rich clinical practice established based on the human body and the laws of nature in the development of thousands of years (127). Chinese medicine often emphasizes disease prevention, encouraging the establishment of healthy lifestyle habits encompassing diet, mood and exercise to improve the body’s immunity and prevent diabetic diseases. Similarly, when managing diseases, such as diabetes, there is an emphasis on preventing their progression. For individuals already living with diabetes, early diagnosis and treatment are crucial to prevent the development of DN, safeguard renal function, and decelerate the progression of diabetes. This approach bears resemblance to modern medical strategies for managing diabetic nephropathy (128).

Mitochondria contain genetic material that produces energy and is involved in various metabolic activities within the cell. Oxidative stress, apoptosis, and inflammatory responses caused by mitochondrial dysfunction (e.g., mtDNA damage, damage to the mitochondrial respiratory chain, altered mitochondrial dynamics, impaired mitochondrial autophagy, and elevated ROS with the onset of oxidative stress) are broadly involved in the development of DN. TCM can play a therapeutic role in DN by regulating the PINK1/Parkin-guided mitophagy pathway, and it can also influence mitochondrial fusion and fission through the regulation of kinetic proteins, such as Mfn1, Mfn2, and Drp1. At the same time, many studies have demonstrated that TCM also reduces the production of ROS, improves the antioxidant capacity of the kidneys, and inhibits oxidative stress in the kidneys.

## 5 Limitations and future prospects

TCM possesses both advantages and limitations in addressing mitochondrial dysfunction in the management of diabetic nephropathy. In terms of safety, compared to certain pharmacological treatments, TCM typically offers enhanced safety and tolerability, rendering it suitable for long-term disease management. Regarding therapeutic stability, TCM often strives to regulate overall balance, thereby modulating the immune system and enhancing the body’s general condition, which contributes to the stability and durability of therapeutic outcomes. As for individualized treatment, TCM emphasizes syndrome differentiation and personalized approaches, enabling the formulation of tailored treatment plans based on the unique physical and medical characteristics of each patient, thereby enhancing the specificity and efficacy of the treatment.

The pathogenesis of DN is multifaceted, often implicating a confluence of factors. Autophagy and pyroptosis are notable contributors to DN, while oxidative stress and inflammatory responses in the kidney have persistently been focal points of research. Emerging studies underscore the roles of ferroptosis (129), gut microbiota (130), myokines (131), immune response (132), and other pathways in DN pathogenesis. These mechanisms may synergistically interact within the renal environment; For instance, DN induced by mitochondrial dysfunction may coincide with the onset of ferroptosis. Regrettably, current research predominantly targets isolated mechanisms, leaving the interplay of multiple pathways in DN insufficiently explored. Additionally, TCM boasts the advantage of a multi-target, multi-pathway, and multi-approach strategy for treating diseases, yet limitations such as low oral bioavailability and unclear target specificity impede drug development. Future advancements must focus on improving technical methodologies, including the extraction and identification technologies of TCM. Compounds and extracts from herbal medicine have demonstrated efficacy in animal models of DN, but the paucity of clinical trials and issues with oral bioavailability constrain their development. Moreover, the complexity of herbal compound identification and the potential side effects of certain TCM components necessitate further experimental validation. In terms of therapeutic strategies, guidance on the combined use of Chinese medicine and modern pharmacotherapy is lacking, necessitating the establishment of standardized protocols for DN treatment. Integrating TCM with nanomedicines to precisely target the kidney and address the issue of low oral bioavailability presents a promising future treatment modality (133). The amalgamation of traditional Chinese and Western medicines may represent the optimal therapeutic approach for DN.

This review encapsulates some of the most promising Chinese medicinal agents for regulating mitochondrial dysfunction in DN treatment. It is anticipated that future advancements in artificial intelligence, coupled with refined identification and extraction technologies for TCM components, will herald high-quality clinical trials focused on mitochondrial dysfunction, thereby elevating the clinical management of DN.

## Author contributions

D-MZ: Writing – original draft, Data curation, Investigation. RZ: Data curation, Investigation, Writing – review & editing. X-TW: Funding acquisition, Writing – review & editing. Z-HY: Writing – review & editing, Funding acquisition.

## Glossary

**Table:**

ACEIs	Angiotensin-converting-enzyme inhibitors
ADP	Adenosine diphosphate
AGEs	Advanced glycosylation end-products
AKAP1	A-kinase anchoring protein 1
AMP	Adenosine monophosphate
AMPK	Adenosine monophosphate-activated protein kinase
ARBs	Angiotensin Receptor Blockers
AS II	Astragaloside II
ATP	Adenosine triphosphate
Bax	Pro-apoptotic protein Bcl-2-associated X protein
Bcl-2	B cell lymphoma 2
BUN	Blood urea nitrogen
CALE	Cassia alata ethanolic leaf extract
CAMK1	Calcium/calmodulin-dependent protein kinase type 1
CAT	Catalase
CKD	Chronic kidney disease
Col1α1	Collagen1α1
ColI	Collagen type I
CPM	Chinese patent medicine
db/db	Diabetic/diabetic
DIO	Diosgenin
DN	Diabetic nephropathy
DNA	Deoxyribonucleic acid
Drp1	Mitochondrial dynamin-related protein 1
ECM	Extracellular matrix
EEL	Ethanol extract of Ligusticum chuanxiong rhizome
EMT	Epithelial-mesenchymal transition
ESRD	End-stage renal disease
Fis1	Mitochondrial fission1 protein
FMN	Formononetin
FN	Fibronectin
GPX	Glutathione peroxidase
GSH-Px	Glutathione peroxidase
GSJD	Gushen Jiedu capsule
HDD	Huangqi Danshen decoction
HG	High glucose
HIF-1α	Hypoxia-inducible factor-1 alpha
HK-2	Human renal proximal tubule
HO-1	Heme Oxygenase 1
HQD	HuangQi decoction
IL-1β	Interleukin-1β
IL-11	Interleukin-11
JAK2	Janus kinase 2
JCYSTL	JinChanYiShenTongLuo Formula
LC3	Microtubule-associated protein light chain 3
MAM	Mitochondria-associated endoplasmic reticulum membrane
MAPK	Mitogen-activated protein kinase
MDA	Malondialdehyde
Mff	Mitochondrial fission factor
Mfn1	Mitochondrial fusion proteins 1
Mfn2	Mitochondrial fusion proteins 2
MnSOD	Mitochondrial superoxide dismutase
MRC	Mitochondrial respiratory chain
mtDNA	Mitochondrial DNA
NADPH	Nicotinamide adenine dinucleotide phosphate hydrogen
nDNA	Nuclear genome
NF-kB	Nuclear factor kappa-light-chain-enhancer of activated B cells
NLRP3	Nucleotide-binding oligomerization domain-like receptor-family pyrin domain-containing 3
NOX4	NADPH oxidase 4
Nrf2	Nuclear factor (erythroid-derived 2)-like 2
OFRs	Oxygen free radicals
OPA1	Optic atrophy protein 1
OS	Oxidative stress
OXPHOS	Oxidative phosphorylation
p53	Cellular tumor antigen p53
PDE4	Phosphodiesterase-4
PGC-1α	Peroxisome proliferator-activated receptor-γ coactivator-1-α
PINK1	Phosphatase and tensin homolog (PTEN)-induced kinase 1
PKA	Protein kinase A
QDTS	QiDiTangShen granules
RASIs	Renin-angiotensin system inhibitor
Rg1	Ginsenoside Rg1
ROS	Reactive oxygen species
SCr	Serum creatinine
SHYS	San-Huang-Yi-Shen capsule
SIRT1	Silent information regulator sirtuin 1
SOD	Superoxide dismutase
STAT	Signal Transducer and Activator of Transcription
STAT 3	Sctivator of transcription 3
STZ	Streptozotocin
TCM	Traditional Chinese medicine
TGF-β	Transforming growth factor β
UA	Uric acid
UACR	Urinary albumin-creatinine ratio
UAlb	24-h urinary albumin
α-SMA	α-smooth muscle actin
